# Vector fields as a framework for modelling the mobility of commodities

**DOI:** 10.1371/journal.pone.0340109

**Published:** 2026-03-13

**Authors:** Sima Farokhnejad, Angélica S. da Mata, Mariana Macedo, Ronaldo Menezes

**Affiliations:** 1 Department of Computer Science, University of Exeter, United Kingdom; 2 Department of Physics, Federal University of Lavras, Minas Gerais, Brazil; 3 Department of Data Science, Northeastern University, London, United Kingdom; 4 Khoury College of Computer Sciences, Northeastern University, Boston, United States of America; 5 School of Computer Science and Engineering, Constructor University, Bremen, Germany; INRAE: Institut National de Recherche pour l'Agriculture l'Alimentation et l'Environnement, FRANCE

## Abstract

Commodities flow through trade networks across the world, with trajectories that can be effectively modelled using approaches similar to those used in human mobility studies. Yet, documenting these movements comprehensively is challenging due to data sparsity, cost, and privacy constraints. Origin-destination (OD) matrices provide a widely used framework for representing mobility, although they inherently omit locations not directly observed as either origins or destinations. This incompleteness creates gaps across different geographical scales, constraining our ability to characterise movement patterns in underrepresented areas. In this study, we introduce a vector-field-based method to address these persistent data challenges. By transforming OD data into continuous vector fields, we capture spatial flow patterns more comprehensively than traditional network approaches, while also enabling robust analysis of mobility directions. Our approach incorporates interpolation techniques that handle incomplete and sparse datasets effectively; when approximately 500 out of 853 areas are removed, 189 areas (36%) maintain degree deviations of less than 15 degrees, showing that the general direction of flow is preserved for over one-third of the impacted areas and enabling continuous spatial analysis. We apply this framework to cattle trade data from Minas Gerais, Brazil. Cattle movements are particularly significant as they directly impact disease transmission, including foot-and-mouth disease. Accurately modelling these flows supports effective disease surveillance and preparedness, with benefits for both animal health and economic stability. Our analysis reveals distinct spatial clusters of trade behaviour, temporal patterns in flow directions, and seasonally varying critical points likely associated with known periodicities in cattle trade driven by breeding cycles, slaughter schedules, and fluctuations in global demand. While previous vector-field studies focused on human mobility, our framework addresses the distinct challenges of commodity flows, where aggregated OD data, sparse observations, and lack of data are the norm. It enables inference in unobserved areas which is a critical capability for modelling scenarios such as disease spread. This approach enhances our capacity to infer flow patterns from incomplete datasets and advances understanding of large-scale commodity trade dynamics.

## Introduction

Mobility pattern modelling has long been the focus of many studies [1–5], with network-based methods playing a central role in capturing the flow of commodities across the world. While networks have proven useful in many contexts, they often miss key nuances, particularly when applied to large, complex and often incomplete datasets. Mobility datasets of commodities, such as those tracking livestock trade, tend to be vast, containing many individual records [6], which makes network generation both computationally expensive and the resulting networks difficult to visualise and analyse [7].

These datasets are also subject to uncertainty [8,9], not only because of errors in data collection but also because it is practically impossible to capture every detail of large-scale movements completely. This uncertainty is inherent to the nature of mobility data [10]. For instance, most work in human mobility relies on datasets that capture only a fraction of reality, such as call detail records (CDRs), extended detail records (XDRs), and location-based social networks (LBSNs) [11,12]. As a result, modelling with incomplete data can introduce inaccuracies and uncertainties in contexts such as urban system analyses or disease spread prediction. Addressing these issues of missing and incomplete data is therefore essential for enhancing the accuracy of mobility modelling and related applications.

Another common limitation is that network models typically rely on origin-destination (OD) matrices, which focus on the starting and ending points of movements [13]. However, this representation is incomplete by design: locations that are not explicitly recorded as origins or destinations are ignored, even though they may play significant roles in the overall flow of commodities. For example, intermediate locations through which commodities are transported can influence the dynamics of trade, yet they often remain invisible in OD matrices. This creates gaps in our understanding of movement patterns, particularly in unrepresented locations.

**Fig 1:**
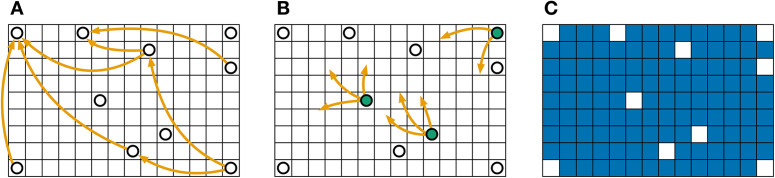
Data sparsity can impact network modelling by disregarding important locations. **(A)** Typical OD model of commodity movements where locations not included in the recorded movements are excluded entirely from the network. **(B)** Certain areas that serve as spatial junctions are omitted simply because they are neither origins nor destinations, despite their potential importance in facilitating flow. **(C)** Data gaps result in missing regions (i.e., blue cells), limiting our ability to assess their significance within the overall movement system. This toy example illustrates how OD models may fail to capture the full spatial complexity of commodity flows.

Take, for example, the scenario in Fig 1. Panel A represents the typical OD model of commodity movements, where only the start and end points are captured. In this representation, certain locations that may serve as critical intersections for multiple movements (illustrated as green nodes in Fig 1B) are not directly represented. Unlike datasets with individual trajectory data that capture consecutive stops, OD matrices omit intermediate locations that could act as important transit points. While these locations are not directly involved in the recorded movements, they may still play an essential role in the overall flow of commodities.

In our modelling, we treat movements as vectors and calculate a resultant vector for each region. We then use interpolation to estimate vectors for regions lacking direct observations (filling the blue cells in Fig 1C). This interpolation is based on the reasonable assumption that neighbouring locations are likely to exhibit similar flow patterns. Our approach thus addresses data gaps by inferring likely flows in unrepresented locations, offering a more comprehensive view of system dynamics. The accuracy and robustness of this interpolation depend on the amount of missing information, as we demonstrate later in Fig 3.

Mazzoli et al. [14] first applied a vector-field framework to human mobility, characterising recurrent commuting flows between home and work in global cities. Using Twitter data and census records, the authors validated their approach and demonstrated that the gravity model with an exponential deterrence function more accurately reproduced both empirical flows and orientations compared to the radiation model. This work introduced a novel theoretical and practical framework for understanding the spatial organisation of urban mobility and supporting urban infrastructure planning.

Liu et al. [15] extended this framework beyond commuting flows to encompass any type of movement, making it applicable to both human and non-human mobility systems. By constructing mesoscopic vector fields directly from trajectory data, they captured both flow intensity and direction, revealed, for example, a systematic imbalance of trips towards city centres, and demonstrated that minimal behavioural rules can reproduce these patterns. This generalised framework has since been adopted in subsequent research, including studies on pedestrian permeability in sidewalk networks [16], new modelling approaches such as opportunity-based and seasonal mobility models [17,18], as well as spatial analytics frameworks [19].

While Mazzoli et al. [14] investigated human mobility and Liu et al. [15] addressed general mobility using complete trajectory or comprehensive commuting data, our work tackles the distinct challenges of commodity flows, which are characterised by aggregated OD data with inherent sparsity and substantial missing information. Our key methodological contributions are threefold. First, we develop robust interpolation techniques based on Delaunay triangulation that reliably infer flow patterns in unrepresented regions, essential for applications such as disease spread modelling, where identifying potential pathways through unmonitored locations is crucial. Second, we introduce entropy and cosine similarity analyses specifically designed to capture the diversity, stability, and temporal dynamics of trade flows, complemented by spatial autocorrelation analysis to uncover regional clustering patterns. Third, we identify critical points (sources and sinks) that reveal the structural organisation of commodity movement networks. Together, these analytical tools, combined with our treatment of sparse aggregated data, distinguish our framework from previous general mobility applications and make it directly applicable to the practical challenges of modelling large-scale commodity systems.

The application of vector fields to commodity flows draws on a mature theoretical foundation established in other domains. In meteorology, for example, vector fields represent wind direction and velocity, providing a rigorous framework to analyse spatially distributed flows and interpolate information where direct measurements are scarce [20]. This theoretical basis makes vector fields particularly suitable for contexts where movements occur over space and time. By translating commodity trade movements into vector fields, we leverage established techniques from weather and fluid flow analysis [21,22], including vector interpolation, field integration [23], and streamline generation [24]. This methodological transfer is not merely an analogy but a theoretically grounded approach that enhances both the accuracy and interpretability of commodity flow analysis, providing insights into spatial dependencies that would remain hidden with purely discrete origin–destination data.

To demonstrate the utility of our approach, we apply this vector field methodology to cattle trade data from the Brazilian state of Minas Gerais, one of the largest cattle-trading regions in Brazil. The scale and complexity of this trade network, coupled with the inherent sparsity of available data, makes it an ideal test case for evaluating our methodology’s effectiveness in transforming OD data into continuous vector fields that capture commodity flow patterns. In traditional models, the lack of data on certain locations limits our ability to predict how disease might spread through areas not represented as origins or destinations. Vector fields, however, allow us to estimate potential livestock flows through these unrepresented areas, offering a more comprehensive tool for assessing epidemic risk. One key application of this method is understanding disease spread in livestock, such as foot-and-mouth disease [25–27].

To analyse the temporal dynamics of commodity flows, we employ entropy [28] and cosine similarity [29]. Entropy quantifies the diversity of trade directions throughout the year, helping to distinguish between regions with stable and fluctuating trade behaviours. Cosine similarity captures the temporal evolution of flow directions across different timescales (e.g., monthly, seasonal, and yearly), allowing us to identify patterns of consistency or variability in trade routes. Beyond these temporal analyses, the vector field approach offers a comprehensive representation of commodity flows at multiple spatial scales. A key aspect involves identifying critical points within the vector field [30], i.e., locations where flows converge (sinks) or diverge (sources), which serve as focal points for understanding both local and global movement structures. We also analyse spatial autocorrelation in flow magnitudes to reveal regional clustering patterns and dependencies. By studying the behaviour and distribution of these critical points, we gain insights into trade dynamics not captured by traditional network models. The classification of critical points provides a structured understanding of the field’s topology and, by assuming smooth transitions between regions, enables construction of a simplified yet informative model of the overall commodity flow system.

Our results drawn from the application of cattle trade in Minas Gerais (Brazil) demonstrate that vector fields offer an efficient and adaptable framework for analysing large-scale commodity mobility datasets.

## Materials and methods

### Data formatting

Our approach for generating vector fields uses origin–destination (OD) data as input, the most common publicly available mobility data format. By segmenting data into appropriate temporal and spatial cells, we project movements onto a grid and generate vectors representing aggregate commodity flows between defined areas. This process is not limited to any single commodity but is applicable to any scenario where movement data can be represented as OD pairs, making it a versatile tool for studying the flow of commodities, goods, or people.

This method is particularly valuable when dealing with imperfect or incomplete information. In many practical situations, certain locations lack explicit records, or data may not cover every possible movement route. The vector field approach addresses these challenges by inferring likely commodity flows and filling spatial gaps using interpolation techniques. Even with partial or uncertain OD data, this methodology offers a comprehensive view of movement patterns, supporting analysis and decision-making across diverse applications. While our methodology is general and applicable to any OD-defined movement data, we focus on cattle trade in the Results section to illustrate the approach.

### Creating vector fields

We transform an origin-destination network into a vector field for the region of interest through the following steps:

**Step 1: Define temporal and spatial granularity.** Choose suitable temporal and spatial granularities, specifying both the timeframe for trades and how the study area is partitioned. Spatial units can be defined using established administrative boundaries (e.g., municipalities) or a grid of regularly sized cells, depending on the aim of the analysis. The spatial divisions may be established units with uneven sizes and shapes, representing various spatial units at different levels of granularity. In this context, they may represent municipalities (Panel A in S1 Fig) and micro-regions (Panel B in S1 Fig) within the state, although higher or lower granularities can be used if needed.

**Fig 2:**
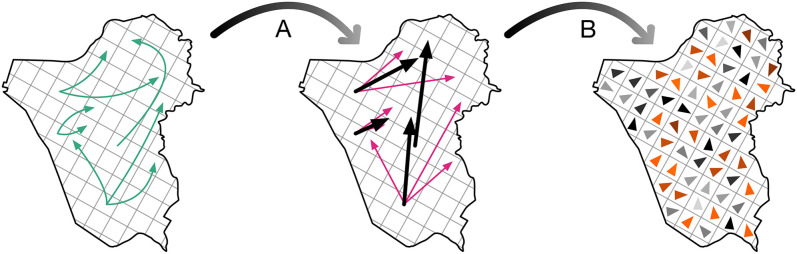
Generating a vector field from origin-destination data. Given locations where we have information (origins/destinations of commodity movements represented by directed arrows) shown in the map on the left, **(A)** we interpret each origin-destination pair as a vector and calculate the resultant vector of all movements from each particular location. Once this is done for all locations with available data, **(B)** we interpolate the missing information, illustrated in the rightmost figure as arrows shown in shades of orange. The final result is a complete vector field where all locations (cities, for instance) have a vector with direction and magnitude. In this example, arrows represent direction and shading represents magnitude. The map used in this figure is freely available (not copyrighted) [31].

**Step 2: Generate vectors from OD matrix units.** Vectors can be generated either for administrative units (e.g., municipalities) or for cell-based grids. Details for municipality- and micro-region-based vector generation are provided in Cattle commodity flows and fields section. For cell-based OD matrices, we project the network (i.e., trades within the chosen timeframe) onto this spatial scheme so that each node lies within a particular cell (leftmost map in Fig 2). For each cell, all outgoing edges are treated as vectors (shown in pink in the middle map), which are combined into a single resultant vector (shown in black) whether through averaging ($\frac{1}{n}\sum_{i=1}^{n}{𝐯}_{i}\;$, S1 Fig), or other relevant methods appropriate to the study. Repeating this process for every cell with outgoing edges produces a partial vector field.

**Step 3: Interpolation to complete the vector field.** As some cells do not generate outgoing edges, we employ interpolation to fill in the missing vectors and complete the full vector field (shown in shades of orange in the rightmost map in Fig 2). Interpolation can be applied using the centroids of unevenly sized administrative units (e.g., municipalities) or centroids of regularly sized cells; the choice depends on the aim of the analysis. Administrative-unit interpolation preserves official boundaries, while cell-based interpolation enables finer-scale flow analysis.

**Step 4: Refinement and critical point detection.** Interpolation can also rescale the vector field to coarser or finer spatial resolutions. For instance, vectors can be interpolated to represent larger units or projected onto a finer grid for detailed analysis. This step is particularly important for detecting critical points, since the choice of cell size must balance resolution and robustness. An optimised cell size (or equivalently, number of cells) is required to ensure reliable detection of critical points (see Section S2 in S1 File for details). An additional consideration is the role of inter-cell trades, particularly when interpolating to larger divisions. In this study, the initial vector field is generated directly at the resolution of the OD matrix units; therefore, inter-cell trades do not occur. After selecting the cell size (or number of cells), the vectors obtained in Step 3 are projected onto this grid. An interpolation method (Section S3) is then applied to estimate a vector for each cell, producing a complete vector field at this resolution, which is subsequently used to identify critical points (Section S6).

We use triangle-based interpolation owing to its simplicity and suitability for sparse networks [32,33]. This method constructs vectors at new points based on known vectors, enabling a continuous representation of commodity flows across the entire region. Technical details on the interpolation method are provided in the Supplementary Materials.

Vector field analysis offers two complementary approaches for understanding commodity mobility patterns: global flow exploration and critical point identification.

Global exploration of flow patterns focuses on how a region’s mobility behaviour changes through its dominant flow vector, as we later demonstrate with cattle trade in Diversity and regularity of (cattle) commodity flows section. This approach examines the vector computed for each region, following the method outlined in Fig 2. Changes in the dominant direction of a region’s vector, as well as similarities between its vector dynamics and those of other regions, can be effectively observed through vector field representations.

Temporal patterns can be quantified using vector directions by applying measures such as cosine similarity and entropy (see Sections S4 and S5 for methodological details), while spatial correlations based on vector magnitudes can highlight groups of cities with comparable trade behaviour, including clusters that reflect different trading distances such as short-, medium-, and long-distance commodity flows. Examining these spatial patterns can identify areas with consistently high or low trade intensity. Comparing these characteristics across multiple time intervals enables clustering of regions with similar dynamic behaviours and identification of key roles in overall mobility patterns.

Critical points in vector fields, where the flow vanishes, reveal essential structural characteristics of mobility patterns. These points can be classified as attracting or repelling based on the eigenvalues of the Jacobian matrix of the field [30]. We illustrate the presence of critical points within a vector field, highlighting regions of flow convergence (see Panel B in S2 Fig). These points are explored in our analysis of cattle trade (see Critical points in cattle vector field section), although their full interpretation in the context of commodity flows remains a question for future research.

In summary, transforming networks into vector fields offers a novel approach to modelling the dynamics of commodity flows. In the context of cattle trade (Results section), this transformation reveals seasonal patterns, evolving critical points, and changes in flow dynamics over time.

## Results

We apply our proposed vector field approach to cattle trade data from Minas Gerais, Brazil (see Section S1 for dataset details) to demonstrate how this method reveals complex mobility patterns. By transforming raw trade data into geographic vectors, we identify key insights such as dominant flow directions, trade hubs, and critical points—insights often missed by traditional network models. Our analysis spans municipality and micro-region levels, using techniques like entropy, cosine similarity, and spatial autocorrelation (see Supplementary Materials for further details). We showcase the method’s ability to handle incomplete data, uncover commodity movement patterns, and support applications such as planning, risk assessment, and disease control.

### Robustness of vector fields

Before analysing the trade data, we assess the robustness of our vector field reconstruction approach by systematically removing spatial regions and evaluating the extent to which original flow directions and magnitudes are preserved. This procedure simulates the presence of missing trade data and demonstrates the capacity of vector fields to interpolate commodity flows in unobserved areas.

**Fig 3:**
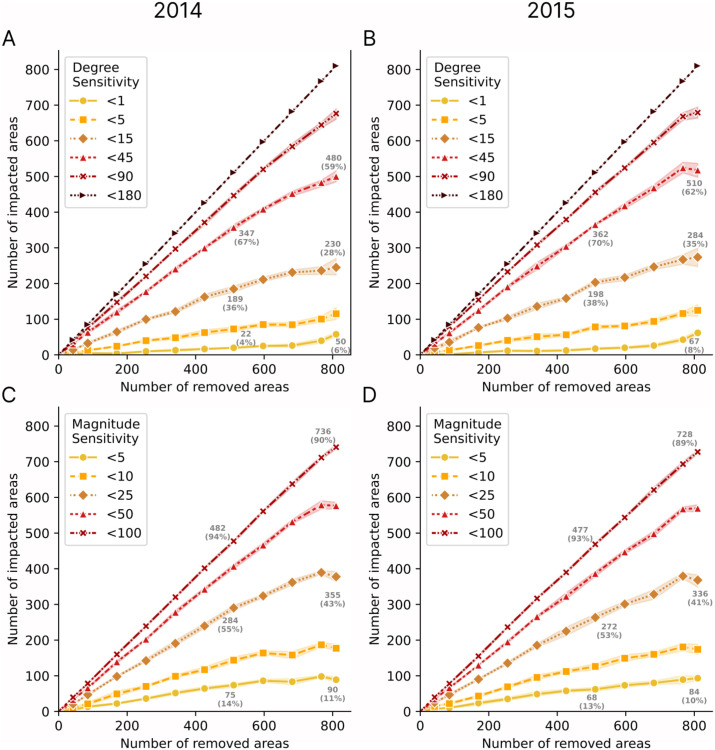
Robustness of vector field directions and magnitudes under spatial data removal. Number of impacted areas as a function of randomly removed areas for 2014 **(A, C)** and 2015 **(B, D)**. Panels A-B show directional robustness, where lines represent the number of areas with angular deviations below specified degree thresholds (< 1^°^, < 5^°^, < 15^°^, < 45^°^, < 90^°^, < 180^°^). Panels C-D show magnitude robustness, where lines represent the number of areas with spatial deviations below specified distance thresholds (< 5 km, < 10 km, < 25 km, < 50 km, < 100 km). Percentages indicate the proportion of areas impacted in relation to the areas removed. The results demonstrate the robustness of vector field estimations despite spatial data gaps, with high consistency between 2014 and 2015, highlighting the reliability of our approach across different years. The total number of areas is 853, and the maximum diameter of Minas Gerais is approximately 1250 km.

As shown in Fig 3, we quantify directional robustness (Panels A and B) and magnitude or distance robustness (Panels C and D) in terms of the proportion of vectors affected and the angular deviation in degrees. Two key findings emerge. First, robustness appears stable across years, with variations typically below 10%. For instance, when approximately 800 areas are removed, 230 areas (28%) in 2014 (Panel A) show deviations below 15 degrees, compared to 284 areas (35%) in 2015 (Panel B). Second, most deviations tend to remain below 45 degrees or 25 km. For example, when approximately 500 areas (60% of 853) are removed in 2014 (Panel A), 347 areas maintain deviations below 45 degrees, indicating that the majority of impacted areas experience directional changes of less than one quadrant. Similarly, for magnitude sensitivity (Panel C), when 500 areas are removed, 284 areas (55%) show distance deviations below 25 km. Given the city-level scale of our analysis, these thresholds constitute relatively minor perturbations. Deviations confined within a single directional quadrant and distances typically under 25 km demonstrate that the reconstruction method can accommodate substantial data gaps while maintaining the integrity of the overall flow structure.

Together, these findings demonstrate that vector flow patterns remain stable across years (comparing Panels A with B, and C with D) and resilient to considerable area removal, with most directional deviations confined within a single quadrant. However, the robustness thresholds established here apply specifically to city-level analysis; finer spatial resolutions would benefit from additional validation to ensure comparable performance at sub-city scales.

Regarding practical applications, our method proves adequate for identifying flow pathways, trade routes, and regional connectivity patterns, even under substantial missing data scenarios. However, applications requiring precise direction and magnitude estimates or fine-grained directional accuracy would benefit from denser spatial coverage. The robustness of sinks and sources under conditions of missing trade data is examined in Section S9, where we confirm that the identification of sinks and sources remains consistent following area removal from the dataset.

### Cattle commodity flows and fields

We use the cattle trade data (described in Section S1) at two levels of granularity: municipalities and micro-regions. For the municipality level (Panel A in S1 Fig), we begin by isolating a single municipality and considering all trades originating from it to others. We do not consider local trades within the same location (self-loops). Each trade is transformed into a vector starting from the centre of the origin and pointing towards the centre of the destination. By averaging these vectors, we obtain a single representative vector, resulting in a partial vector field that captures the predominant flow direction and intensity over a given time period. This process exemplifies how scattered trade data are aggregated into a coherent vector field at the municipal scale.

To complete this representation, we apply triangle-based interpolation as it preserves spatial structure and avoids artefacts by considering local patterns [32,33] (further details in Supplementary Materials). This fills in gaps where direct trade data are absent, creating a complete vector field over the entire municipal region that faithfully represents the continuous flow patterns of cattle movements.

### Diversity and regularity of (cattle) commodity flows

We analyse the monthly vector fields from the trade data for two distinct spatial divisions: municipalities and micro-regions. Our goal is to assess how the directions of these commodity flows evolve over time and across different regions. To achieve this, we use two key metrics:

**Entropy:** measures the diversity of trade directions over the year. In particular, we use Shannon entropy, which quantifies how often trades occur across different directions (see Section S5 for methodological details).

**Cosine similarity:** captures the similarity (or dissimilarity) in commodity flow direction between pairs of consecutive months. A value near *1* indicates that two monthly vectors point in nearly the same direction (high regularity), while a value near −1  indicates they point in opposite directions (high variability).

During each year, every municipality (or micro-region) has 12 monthly vectors of trade flows. We categorise each vector into one of four quadrants based on its direction in the Cartesian plane, thus reducing the original set of 12 vectors to a sequence of 12 quadrant values. We then compute the Shannon entropy of this sequence, capturing the diversity or unpredictability of trade directions over the year.

**Fig 4:**
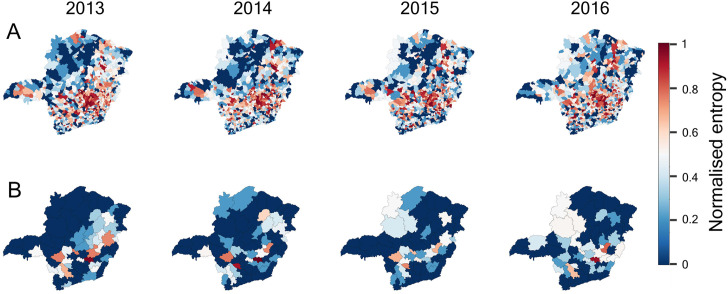
Entropy of monthly vectors for (A) municipalities and (B) micro-regions. Monthly vectors are classified into four directional clusters by dividing the Cartesian plane into four quadrants, each assigned a distinct value (1–4). Shannon entropy is then calculated from these 12 cluster values to yield a single entropy measure per municipality or micro-region, which is colour-coded on the map to indicate the diversity of commodity flow directions over the year. For visualisation, entropy values are normalised using Min–Max normalisation. The base maps used in this figure are freely available and not subject to copyright (Panel A: [34]; Panel B: [35]).

Fig 4 presents entropy heatmaps of municipalities and micro-regions. Many municipalities (and micro-regions) in the North exhibit lower entropy (darker blue shades, closer to zero), indicating more stable trading directions. Some regions with higher entropy (darker red shades) have more frequent shifts in commodity flow direction (the term ‘commodity’ refers to ‘cattle as commodity’ in the Results section given that cattle trade has been used as the case study), indicating greater unpredictability. As the highest inter-regional trade volume in Minas Gerais occurs between the North and West regions, unpredictability is mostly observed in the South-east—regions with less established trading relationships.

**Fig 5:**
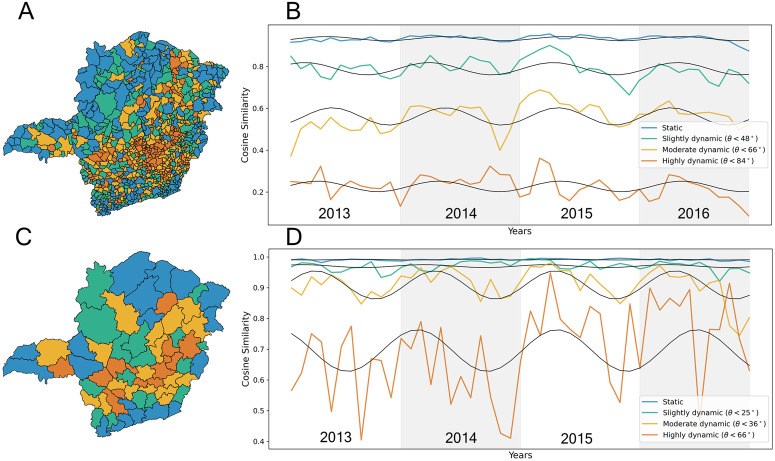
Cosine-based clustering of (A, B) municipalities and (C, D) micro-regions. Using k-medoids clustering (k = 4) (see Section S8 in S1 File) for further explanation), locations are grouped by their cosine similarity values. Clusters, indicated by distinct colours, reveal that in many peripheral locations the commodity flow directions remain stable over the years. Freely available maps are used in this figure (Panel A: [34]; Panel C: [35]).

To assess how trade directions evolve over short time intervals, we use the cosine similarity between pairs of consecutive monthly vectors for each municipality or micro-region (e.g., from January 2013–February 2013 to November 2016–December 2016). This method allows us to focus on the structure and direction of commodity flows rather than their magnitude, making it well-suited for detecting shifts or redirections in trade patterns over time. We then use these cosine similarity values to cluster municipalities and micro-regions based on their month-to-month flow direction patterns (Fig 5), identifying four clusters ranging from highly static (shades of blue) to highly dynamic (shades of orange). In essence, we group areas (cities or micro-regions) whose flow directions not only change month-to-month by similar angles but also follow similar patterns of change throughout the year, capturing both the magnitude and rhythm of directional shifts. Fig 5 displays the average cosine similarity values within each cluster. Detailed cosine similarity values for individual areas are provided in our previous study [36].

In both municipalities (Fig 5A–B) and micro-regions (Fig 5C–D), a large dominant cluster emerges, consisting of areas with relatively stable flow directions (cosine values close to 1), indicating regular and predictable cattle movement. However, there are cities and micro-regions where flow directions vary significantly, with changes more than 45 degrees across several monthly intervals over the four-year period. Even in these more dynamic cases, the angular changes exhibit a periodic, sinusoidal-like pattern, with a noticeable drop in cosine similarity, indicating increased angular change, towards the end of each year (e.g., November–December), as shown in Fig 5B and 5D.

This observed rhythmicity reveals a deeper temporal structure in trade patterns, going beyond static flows or random fluctuations. Even if the peak of directional change shifts in time across regions (e.g., occurring earlier or later in the year), the underlying wave-like structure remains, highlighting the existence of region-specific temporal rhythms. These findings indicate a higher level of predictability in cattle movement dynamics and demonstrate that the combined use of entropy (to measure directional diversity) and cosine similarity (to assess regularity) can be effectively extended to analyse specific types of cattle trade transactions.

### Going beyond direction in vector fields

In our previous findings, we focused primarily on the direction of vector fields. While this provided valuable insights, it overlooked a key dimension: magnitude. Our methodology calculates individual vectors from the geographical coordinates (latitude and longitude) of origin-destination pairs, where each vector’s magnitude directly represents the Euclidean distance between locations. When we average these vectors for each municipality (as illustrated in Panel A in S1 Fig), the resulting magnitude reflects both the predominant direction and the characteristic distance of trades from that location. Two vectors may share the same direction yet represent very different dynamics, with one indicating a short trade distance and the other a much longer one. By incorporating vector magnitudes into our analysis, we expand the study to capture both spatial and temporal variations in trade distances, offering a more comprehensive view of mobility patterns. Specifically, we investigate whether regions tend to have trade distances similar to those of their neighbouring areas. This spatial analysis sheds new light on the role of geographic proximity in shaping trade behaviour and commodity movement patterns.

**Fig 6:**
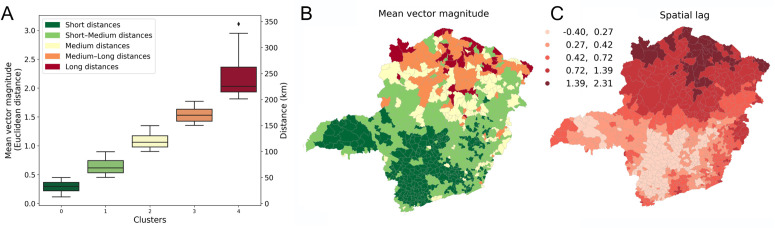
Distribution and maps of trading distances and spatial lag values. **(A)** Municipalities are grouped into five predefined distance-based clusters: short (< 50 km), short–medium (50–100 km), medium (100–150 km), medium–long (150–200 km), and long (>200 km). For each municipality, the trade vector magnitude was computed as the mean of all monthly vectors across four years. The *x* and *y* components of the vectors were computed as the Euclidean differences between the latitude and longitude coordinates of the points and then converted into distance values (km). The distributions of vector magnitudes and distances for each cluster are shown. **(B)** Map of municipalities coloured by distance-behaviour cluster, with trade vector magnitudes and distances represented as in **(A)**. **(C)** Spatial lag values of the vector magnitudes in **(B)**, calculated using Queen-based spatial weights. These highlight municipalities whose values differ from their neighbours, revealing patterns such as ‘doughnuts’ (low values encircled by high ones) and ‘diamonds’ (high values encircled by low ones). Spatial lag values are grouped into five ranges based on the boxplot distribution of all 853 municipalities. The base maps in this figure are freely available (not copyrighted) [34].

Fig 6 illustrates the spatial variation in vector magnitudes across municipalities and the corresponding spatial lag values. These maps highlight municipalities with significantly higher or lower vector magnitudes than their neighbours, identifying notable patterns such as ‘doughnuts’ (low values surrounded by high values) and ‘diamonds’ (high values surrounded by low values). These features are particularly evident in the spatial lag map (Fig 6C), which provides a more distinct representation of clusters compared to the raw vector magnitude map (Fig 6B).

**Fig 7:**
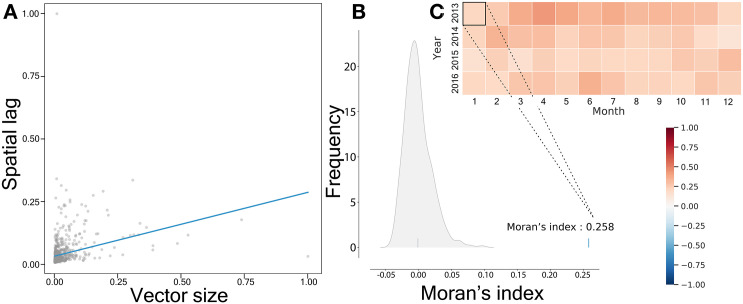
Global Moran’s *I* calculation. **(A)** Scatter plot of vector magnitudes against spatial lag with a fitted line. **(B)** To evaluate the significance of the observed pattern, we compared the Moran’s value calculated from the vector field to those from 1000 simulations in which vector magnitudes were randomly shuffled among municipalities. The observed Moran’s value of 0.258 was significantly higher than the simulated values, indicating a non-random spatial structure. **(C)** The heatmap shows Moran’s values for monthly vector fields across different years. Since no blue shades appear in the heatmap, all calculated Moran’s *I* values are positive, suggesting positive spatial autocorrelation, meaning that municipalities with high vector magnitudes tend to be located near each other.

To quantify spatial relationships, we compute Moran’s *I*, a global measure of spatial autocorrelation (see Section S7). The Moran scatter plot (Fig 7A) reveals a positive correlation between vector magnitudes and their spatial lags, indicating a tendency for similar values to cluster spatially. The observed Moran’s *I* value (Fig 7B) is statistically significant, confirming that vector magnitudes are not randomly distributed—also visually evident in Fig 6. This spatial clustering persists over time, as shown in the Moran’s *I* heatmap (Fig 7C), which highlights consistent autocorrelation across monthly vector fields.

By analysing vector magnitudes alongside directions, we reveal deeper patterns in trade dynamics and regional differences. While demonstrated here with cattle trade, this vector field approach is broadly applicable to other commodity flows, offering valuable insights for logistics planning and policy development.

### Critical points in cattle vector field

To uncover dynamic movement patterns, we use interpolation methods to estimate vectors between known points, transforming scattered data into continuous vector fields. To find critical points, the vector fields are generated using division into cells rather than relying solely on municipality divisions. These fields not only capture the flow of commodity trade but also reveal regions of attraction and repulsion, offering valuable insights into spatial dynamics, particularly for understanding the spread and containment of epidemics. By analysing our vector field data, we identify areas where trade concentrates (e.g., high-risk zones for epidemics) and where it diverges (e.g., key suppliers or hubs that can facilitate disease spread).

**Fig 8:**
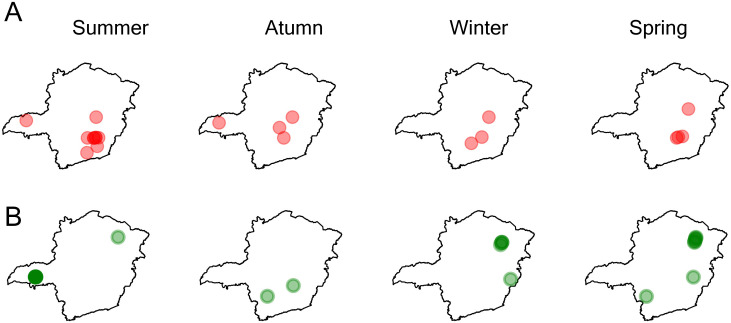
Sinks (A) and sources (B) in the cattle trade vector field. Initial vector fields were generated from cattle trades in each season of 2016. A triangle-based interpolation method is used to generate a continuous vector field from these initial vectors. Critical points within the field are identified, distinguishing attracting points (sinks) from repelling points (sources). Circles indicate the locations of these points, with larger circles used for individual points to enhance visualisation, and darker shading representing higher numbers of points. The boundary maps shown in this figure are freely available (not copyrighted) [37].

To quantify these patterns, we calculate the critical points in our seasonal vector fields in 2016, distinguishing attracting points (sinks, shown in Fig 8A) from repelling points (sources, shown in Fig 8B). The locations of these sinks and sources shift across seasons, suggesting that while some regions maintain directional stability over time (Fig 5), disease outbreak management requires careful consideration of the specific month, year, and trade dynamics as these critical points evolve throughout the period. In Section S9, we show that sinks and sources remain robust to area removal from our dataset.

The movement of cattle follows a structured network, where seasonal shifts and geographical factors shape the distribution of sources and sinks. This variability reflects a combination of factors, including regional geography, the type of premises (e.g., farms, slaughterhouses, or rearing centres), the purpose of trades, and broader seasonal trends in cattle trade. For instance, source areas become more prominent during breeding seasons, such as spring and summer [38], while a high density of sinks corresponds to periods of higher cattle slaughter, particularly in the New Year period. During this period, sources are also more noticeable (darker green in the north-east). In addition, we observe that regions with a high density of slaughterhouses (reported for 2016 [39]) overlap with high-density sink areas (darker red) in our vector field data during spring and summer.

These patterns highlight not only the mobility within the trade system but also the changing roles of different regions in the commodity flow network. Over time, the distribution and strength of sinks and sources may be tied to evolving logistical strategies, shifting market demands, and the structural importance of specific regions. The northern regions frequently act as sources, likely serving as hubs for long-distance cattle movements. The seasonal vector fields represent cattle movements rather than fixed farm locations. Given the considerable distance between the northern and southern parts of the state, cattle are typically routed through the central-eastern regions, which act as intermediate hubs before reaching their final destinations. A comparison of the maps in Fig 8 and Fig 6B reveals that sink areas often coincide with short- to medium- distance transport zones, while medium- to long-distance transport regions are typically associated with source areas.

## Discussion

This study introduces a vector-field-based framework for analysing commodity mobility, which bridges gaps in traditional origin-destination (OD) approaches by incorporating unrepresented locations into a comprehensive view of mobility patterns. Using cattle trade in the state of Minas Gerais, Brazil, as a case study, we demonstrate that vector fields are an effective tool for revealing dynamic trade patterns, including: *(i)* clusters of stable trade direction over time, *(ii)* spatial autocorrelation in commodity flow magnitudes, and *(iii)* key attractors and repellers within the region.

Vector fields provide a comprehensive framework for representing flows, capturing both intensity and direction simultaneously, enabling interpolation in regions with missing data, and supporting advanced analyses such as critical-point identification, temporal patterns, and spatial autocorrelation. While alternative methods (such as origin-destination matrices or trajectory-based models) can represent flow direction, they often cannot reconstruct flows in unobserved regions or require detailed individual-level data. Our approach combines these strengths in a continuous, scalable, and theoretically grounded framework. Limitations include sensitivity to the density and quality of input data, potential artefacts in sparsely observed regions, and the focus on a single commodity in this study (see Section S10 in S1 File for a detailed discussion of methodological limitations and assumptions). Although based on widely used origin–destination data, the method can be readily adapted to other sources, such as CDR or XDR records. Future work could explore higher-resolution data, multi-commodity flows, and applications to human mobility, further extending the versatility and impact of the vector-field framework.

It should be noted that the application of vector fields to human mobility is not entirely novel. Previous works, such as Mazzoli et al. [14] and Liu et al. [15], introduced and generalised vector-field frameworks for characterising recurrent commuting flows and broader mobility patterns from individual trajectories. Our approach builds on this foundation but focuses on commodity mobility using OD matrices, incorporating robust interpolation to infer flows in unrepresented locations, and leveraging entropy and critical-point analysis (sources and sinks) to capture the diversity, stability, and structural patterns of trade flows.

A key contribution of this work is the development of robust interpolation techniques that address incomplete and sparse datasets, enabling continuous mobility analysis across spatial regions. Our robustness analysis demonstrates that vector flow remains stable across years and largely unaffected by substantial area removal, with most directional changes confined within a single quadrant and magnitude deviations typically below 25–50 km. These findings suggest that the framework can be applied confidently in scenarios with incomplete data coverage, such as regions with limited surveillance infrastructure or privacy-restricted datasets. Sources and magnitude show greater sensitivity to area removal, as they are directly affected by data reduction. This highlights that the primary utility of vector flow analysis lies in identifying directional patterns and the structural organisation of flows, rather than providing precise quantification of trade distances (magnitude). For applications requiring accurate magnitude estimation, denser data coverage is recommended.

Additionally, the introduction of entropy and cosine similarity analyses derived from vector fields offers novel ways to measure trade diversity, predictability, and month-to-month flow dynamics. Entropy quantifies the variation in trade directions, identifying regions with stable or unpredictable patterns, while cosine similarity captures temporal changes in flow dynamics, enabling the clustering of regions with similar trade behaviours. Building upon this, we extend our analysis beyond directional components to incorporate vector magnitudes, which reflect the characteristic trade distances from each location. By integrating both direction and magnitude, we reveal how spatial proximity and trade distance jointly influence mobility patterns. This dual-dimensional approach provides a richer understanding of how regions differ not only in where they trade but also in how far commodities typically travel, unveiling spatial clusters. These analytical tools enhance our understanding of spatial and temporal dimensions of commodity flows.

Another important contribution of this work is its compatibility with established vector field theories, which open avenues for advanced analyses. Techniques such as critical point dynamics analysis could be explored to understand the temporal evolution of sinks and sources, or streamline visualisation could be applied to trace continuous trade routes. These analytical approaches enable a more granular understanding of mobility patterns and facilitate predictions about unobserved areas based on inferred commodity flows.

By transitioning from static network representations to dynamic vector fields, this research offers a versatile and scalable framework for studying commodity mobility, advancing our understanding of spatial flows and establishing a foundation for future studies to refine and expand applications in diverse fields, from public health to economic geography.

Critical points, such as sinks, sources, and saddle points, provide deeper insights into the structural dynamics of commodity flows by identifying regions where trade activity converges, diverges, or transitions. Incorporating the identification of these points into the vector field framework enhances our understanding of how mobility patterns evolve over time and space, offering more detailed information about trade hubs, bottlenecks, and areas of influence. This analysis is particularly relevant for understanding how trade dynamics may impact broader systems, such as disease spread, resource allocation, or regional economic stability. Tracking the temporal evolution of these points could provide valuable insights into the stability and resilience of trade networks under changing conditions, such as seasonal variations or economic disruptions.

Our findings highlight strong temporal variability in the spatial distribution of sink and source regions within the cattle trade system. Not only do the locations of these critical points shift across seasons, but their intensity also varies, as seen in the fluctuating density patterns in Fig 8. This temporal variation likely reflects the evolving roles of different premises and regions across seasons, influenced by market-driven dynamics and biological cycles such as breeding [38]. For instance, some premises may act as sources during active breeding or fattening periods, while others, such as slaughterhouses, consistently act as sinks due to receiving cattle without sending them onwards. Notably, these critical points may sometimes emerge in regions not directly present in the observed origin–destination data, suggesting that depending on the commodity type or trade characteristics, influential areas may arise that are not explicitly recorded in the original dataset.

This seasonal variability could also reflect larger economic or logistical factors (e.g., transport infrastructure or policy changes) that influence the movement of cattle over time. Future research could explore how different modes of transportation or changes in trade regulations might influence the emergence, persistence, or reconfiguration of these sinks and sources throughout the year, and how the characteristics of critical points (i.e., their location and movement) relate to the underlying drivers of trade, enabling targeted interventions or policy decisions.

The analytical framework presented here offers several practical implications for policy and intervention strategies in commodity trade systems. The identification of persistent sink regions, for instance, highlights locations where commodities consistently accumulate, making them strategic points for surveillance or inspection programmes. In the context of livestock trade, such regions could be prioritised for disease monitoring or biosecurity measures, as they represent convergence points where animals from diverse origins come into contact. Conversely, regions identified as stable sources could be targeted for compliance programmes or quality assurance initiatives, particularly if they consistently supply large volumes to the broader network. The entropy analysis provides additional policy value by identifying regions with unpredictable trade patterns, which may require more flexible or adaptive monitoring approaches compared to regions with stable, predictable flows. Furthermore, tracking the temporal evolution of critical points enables policymakers to anticipate seasonal shifts in trade dynamics and adjust resource allocation accordingly. For example, if sink regions shift predictably with breeding cycles, surveillance infrastructure could be deployed dynamically rather than maintained year-round at fixed locations. The cosine similarity clustering also reveals regions with similar temporal trade behaviours, suggesting that interventions designed for one region within a cluster might be effectively adapted to others. Importantly, the framework’s ability to infer flows in unobserved regions allows for risk assessment even in areas with limited direct data, though such inferences should be interpreted cautiously and validated where possible. These applications extend beyond disease control to broader contexts such as market regulation, supply chain optimisation, and environmental impact assessment, where understanding the spatial and temporal structure of commodity flows is essential for effective governance.

## Supporting information

S1 File Supplementary Materials for the manuscript: Vector fields as a framework for modelling the mobility of commodities. (PDF)

S1 Fig Toy example illustrating the generation of vector fields from cattle trade data for (A) municipalities and (B) micro-regions. **(A-1)** and **(B-1)** illustrate trade flows originating from a grey part (municipality or micro-region) to others. **(A-2)** and **(B-2)** represent the transformation of these trades into vectors, drawn from the centre of the grey area to the centres of destination areas, and then aggregated into a single resultant vector (shown in red). **(A-3)** and **(B-3)** demonstrate complete vector fields, where interpolation has been used to estimate vectors for municipalities or micro-regions lacking trade data during the selected time window. Different colours indicate varying vector magnitudes. This approach is applied to a specific time window and geographic division but can be adapted to different temporal or spatial granularities depending on analytical needs. Base maps used in this figure are freely available (Panel A: [34]; Panel B: [35]).
(TIFF)

S2 Fig Critical points in the vector field. (A) Triangle-based interpolation method. To estimate vectors at specific locations within the triangulated mesh (generated using Delaunay triangulation on points with known vectors), we use a triangle-based interpolation technique. This method calculates the vector at a point by using the vectors at the three surrounding vertices. (B) Interpolated vector field. The resulting field displays interpolated vectors, with grey regions indicating critical points, which represent areas of attraction or repulsion. Colour variations reflect differences in vector magnitudes. The map used in this figure is freely available (not copyrighted) [31].
(TIFF)

S3 Fig Vector field structure of attracting and repelling critical points. For each type, the characteristics of the Jacobian eigenvalues (real and imaginary parts), as well as the determinant and trace of the Jacobian matrix, are stated above the corresponding field structure.
(TIFF)

S4 Fig K-medoids clustering configuration. (A) Cluster validity curve based on the within-cluster sum of squared distances (commonly referred to as the ‘elbow method’), used to estimate the optimal number of clusters for municipalities and micro-regions.**(B)** Gap Statistic curve, used to validate the estimated number of clusters and assess clustering stability for municipalities and micro-regions, with a dashed line indicating the optimal number of clusters.
(TIFF)

S5 Fig Robustness of where the sinks and sources are calculated while removing a percentage of areas. The y axis shows the relative distance between the original sinks and sources in comparison to the ones using the entire dataset.
(TIFF)
