# Supplementary material for: Vector fields as a framework for modelling the mobility of commodities
Source: PLoS One. 2026 Mar 13;21(3):e0340109. doi: 10.1371/journal.pone.0340109 (PMC12987471; doi:10.1371/journal.pone.0340109)
Supplement: S1 File — (PDF) [file pone.0340109.s001.pdf]

Supplementary Materials for the manuscript: Vector fields as a framework for modelling the mobility of commodities

Sima Farokhnejad, Angélica S. da Mata, Mariana Macedo, and Ronaldo Menezes

S1 Dataset and code availability

This study examines cattle movement in Minas Gerais, Brazil, using data from the Institute of Agriculture (IMA) covering 2013–2016. The dataset includes approximately 420,000 locations (e.g., farms, slaughterhouses, and markets) and records 3.78 million transactions for purposes such as fattening, breeding, and trade. Each movement details the origin, destination, species, gender, age, and number of animals. Import and export data (less than 5% of transactions) were excluded to focus on internal movement patterns. For the analyses presented in this study, origin-destination (OD) matrices (both monthly and seasonal) were used. These, together with all the code for analysis and visualisation, are available at `https://github.com/Sima-Far/Flow_vector_field`.

Fig S1: **Toy example illustrating the generation of vector fields from cattle trade data for (A) municipalities and (B) micro-regions.** **(A-1)** and **(B-1)** illustrate trade flows originating from a grey part (municipality or micro-region) to others. **(A-2)** and **(B-2)** represent the transformation of these trades into vectors, drawn from the centre of the grey area to the centres of destination areas, and then aggregated into a single resultant vector (shown in red). **(A-3)** and **(B-3)** demonstrate complete vector fields, where interpolation has been used to estimate vectors for municipalities or micro-regions lacking trade data during the selected time window. Different colours indicate varying vector magnitudes. This approach is applied to a specific time window and geographic division but can be adapted to different temporal or spatial granularities depending on analytical needs. Base maps used in this figure are freely available (Panel A: [1]; Panel B: [2]).

S2 Cell size selection and intra-cell flow

In step 4 of the methodology, when generating vector fields at a resolution finer than the OD grid, cell size must be selected carefully. Our first criterion is that cell size should be no larger than half of the minimum distance between OD nodes [3], ensuring that each grid cell contains at most one OD node. The second criterion arises from the limitations of the triangular interpolation method: it is highly sensitive to grid resolution. In regions with high-magnitude vectors, linear interpolation over large triangles can result in undersampling and fail to capture fine-scale variations. To reduce this error, the grid cells must be small enough to resolve the most rapid spatial changes. As a partial solution, we introduced a maximum threshold for grid cell size, set to the inverse of the largest vector magnitude in the dataset ($\frac{1}{\|\mathbf{v}_{\text{max}}\|}$). In practice, we combine the two criteria by selecting the minimum of (i) half of the smallest OD node distance ($\frac{1}{2}\min(d_{ij})$, where d_{ij} is the Euclidean distance between the centres of municipalities in our study), and (ii) $\frac{1}{\|\mathbf{v}_{\text{max}}\|}$, and then compute the number of grid cells as $N = \frac{L}{h}$, where L is the map width and h is the chosen cell size.

While the $\frac{1}{\|\mathbf{v}_{\text{max}}\|}$ criterion can be computationally demanding when vectors are very large, alternative approaches have been proposed in the literature, such as setting the threshold $h \leq \frac{1}{2}\min(d_{ij})$ or applying the Nyquist-type criterion, $h \leq \frac{1}{2f_{\text{max}}}$ [4] (where f_{max} is the maximum observed gradient of the vector field). Once a cell size is fixed, the robustness of results can be tested by halving or doubling N.

If the goal is to perform interpolation at a coarser resolution (step 4), it is important to consider the potential role of intra-cell trades. In this study, the initial vector field was generated directly at the resolution of the origin-destination (OD) matrix units; therefore, inter-cell flows do not occur. When interpolating vectors to grid cells in order to produce a continuous vector field for critical point analysis, the grid size is selected (as described earlier) such that each cell contains at most one OD unit (municipality centre point in our case). This ensures that no intra-cell flows need to be considered.

S3 Interpolation of vector fields

There are multiple interpolation methods available for developing a continuous vector field. Among these, we opt for triangle-based interpolation due to its computational efficiency and its suitability for handling the sparsity of our dataset's networks. Triangle-based interpolation enables us to estimate vector values at grid points where no initial vectors are present, whilst Delaunay triangulation efficiently manages sparse and irregularly spaced data by leveraging the geometric relationships between existing points [5], ensuring a smooth and coherent representation of commodity flows.

To perform the interpolation, we use the centres of cells with initial vectors, along with selected boundary points assigned zero vectors to mitigate boundary effects. First, we triangulate the region using Delaunay triangulation on these points, ensuring that each point of interest lies within a well-defined triangular structure. Assume point p falls inside a triangle with vertices having initial vectors $\mathbf{v}_1$, $\mathbf{v}_2$, and $\mathbf{v}_3$, as illustrated in Fig S2A. In our initial implementation, the interpolation weights were defined using the perpendicular distances h_1, h_2, and h_3 from point p to the sides opposite the vertices with vectors $\mathbf{v}_1$, $\mathbf{v}_2$, and $\mathbf{v}_3$, respectively. The interpolated vector at point p was then given by [5,6] $\mathbf{v}_p = \frac{h_1}{h_1+h_2+h_3}\mathbf{v}_1 + \frac{h_2}{h_1+h_2+h_3}\mathbf{v}_2 + \frac{h_3}{h_1+h_2+h_3}\mathbf{v}_3$.

Whilst this height-based formulation is intuitive, it can suffer from numerical instability when triangles are very narrow or nearly degenerate, since dividing by small side lengths amplifies rounding errors. In practice, this occasionally produced a few very large vectors compared to the surrounding field, introducing artefacts into our results. To address this, we adopted an alternative area-based formulation, where the weights are defined as the relative areas of the sub-triangles A_1, A_2, and A_3 formed between point p and the edges of the enclosing triangle. In this approach, the interpolated vector is computed as

$$\mathbf{v}_p = \frac{A_1}{A_1 + A_2 + A_3}\mathbf{v}_1 + \frac{A_2}{A_1 + A_2 + A_3}\mathbf{v}_2 + \frac{A_3}{A_1 + A_2 + A_3}\mathbf{v}_3. \quad \text{(S1 Eq)}$$

This area-based barycentric interpolation is scale-invariant, more robust to skinny triangles, and mathematically equivalent to computing barycentric coordinates using triangle sub-areas. It is a well-established technique in computational geometry and finite element methods [7].

Although the area-based formulation is more robust, numerical issues can still arise if $A_1 + A_2 + A_3$ becomes very small (e.g., when p lies almost exactly on a vertex or the triangle is nearly degenerate). To mitigate this, we introduce a small threshold parameter in our interpolation method that is $\varepsilon = 10^{-12}$: if $A_1 + A_2 + A_3 < \varepsilon_{\text{th}}$, we simply assign $\mathbf{v}_p$ as the average of the vertex vectors $(\mathbf{v}_1 + \mathbf{v}_2 + \mathbf{v}_3)/3$. This safeguard eliminates spurious large values whilst preserving the overall smoothness of the interpolated field.

Fig S2: **Critical points in the vector field.** **(A)** Triangle-based interpolation method. To estimate vectors at specific locations within the triangulated mesh (generated using Delaunay triangulation on points with known vectors), we use a triangle-based interpolation technique. This method calculates the vector at a point p by using the vectors at the three surrounding vertices. **(B)** Interpolated vector field. The resulting field displays interpolated vectors, with grey regions indicating critical points, which represent areas of attraction or repulsion. Colour variations reflect differences in vector magnitudes. The map used in this figure is freely available (not copyrighted) [8].

This interpolation method ensures that the resulting vector field is both continuous and adaptive to the underlying network structure. The final vector field representation, illustrated in Fig S2B, captures both the main flows for each cell and the transitions across the entire field, offering a more detailed spatial understanding of commodity movements.

S4 Cosine similarity

To quantify temporal patterns in flow directions, we use the cosine similarity measure, which quantifies the directional consistency of commodity flows over time. For region k between time intervals i and j, the similarity is given by [9]:

$$S_C(\mathbf{v}_k^i, \mathbf{v}_k^j) = \frac{\mathbf{v}_k^i \cdot \mathbf{v}_k^j}{\|\mathbf{v}_k^i\|\|\mathbf{v}_k^j\|}, \quad \text{(S2 Eq)}$$

which represents the dynamic auto-correlation of the flow direction for region k. A value close to 1 indicates high directional stability over time, whilst lower values suggest greater variability. The procedure consists of the following steps:

1. Generating multiple vector fields over predetermined time intervals (e.g., months over four years).
2. Computing cosine similarity values between consecutive intervals for each region.
3. Constructing feature vectors for each region based on these similarity values.
4. Applying clustering methods to group regions with similar temporal mobility patterns.

This approach allows us to categorise regions based on the stability and evolution of their commodity flow directions, offering insights into the persistence and variability of trade movement patterns.

S5 Shannon entropy

Shannon entropy is a measure of uncertainty or randomness associated with a set of possible outcomes. In a physical sense, it quantifies how dispersed or unpredictable a system is, akin to the concept of disorder in thermodynamics. It can be applied to assess the variability within a vector of values, capturing how evenly distributed different outcomes are [10]. Given a vector $\mathbf{x} = [x_1, x_2, \ldots, x_n]$ where each x_i represents a category, event, or observed outcome, we first convert these values into a probability distribution. Let p_i denote the probability (or relative frequency) of outcome i occurring in the vector. The Shannon entropy $H(\mathbf{x})$ is then defined as [11]:

$$H(\mathbf{x}) = -\frac{1}{\log n} \sum_{i=1}^{n} p_i \log p_i \,, \qquad \text{(S3 Eq)}$$

where the logarithm is typically taken in base 2 (yielding entropy in bits). This equation sums over all possible outcomes, weighting each by the negative logarithm of its probability. Higher entropy indicates greater diversity or unpredictability in the outcomes represented by the vector, meaning that commodity flow directions are more evenly distributed across different routes. Conversely, lower entropy suggests a more regular or predictable pattern, where flows concentrate in fewer, more predictable pathways. This process yields a single scalar value $H(\mathbf{x})$ that summarises the diversity of the vector's components.

S6 Critical points

Critical points in a vector field are essential for understanding the field's structure and dynamics. These are points where the magnitude of the vector field becomes zero, i.e., $\mathbf{v}(\mathbf{x}) = 0$, meaning all components of the vector vanish. In two-dimensional fields, these points are locations where field lines asymptotically converge or diverge, making the vector field's direction indeterminate.

A simple critical point is a special case where the vector field magnitude is zero at the point, but it does not vanish in its immediate surroundings. The behaviour near critical points can be studied using local linear approximations, assuming the vector field is smooth and differentiable. By employing a Taylor series expansion around a critical point $\mathbf{x}_0$, the local behaviour can be expressed as [12]:

$$\mathbf{v}(\mathbf{x}) = \mathbf{v}(\mathbf{x}_0) + \frac{\partial \mathbf{v}}{\partial \mathbf{x}}(\mathbf{x} - \mathbf{x}_0) \,. \qquad \text{(S4 Eq)}$$

Because $\mathbf{x}_0$ is a critical point, $\mathbf{v}(\mathbf{x}_0) = 0$. Equation S4 Eq can then be rewritten in matrix form as follows:

$$\begin{bmatrix} v_x \\ v_y \end{bmatrix} = \begin{bmatrix} \frac{\partial v_x}{\partial x}(x_0, y_0) & \frac{\partial v_x}{\partial y}(x_0, y_0) \\ \frac{\partial v_y}{\partial x}(x_0, y_0) & \frac{\partial v_y}{\partial y}(x_0, y_0) \end{bmatrix} \begin{bmatrix} x - x_0 \\ y - y_0 \end{bmatrix} \,, \qquad \text{(S5 Eq)}$$

$$\mathbf{v} = \mathbf{J} \cdot (\mathbf{x} - \mathbf{x}_0) \,. \qquad \text{(S6 Eq)}$$

The Jacobian matrix, $\mathbf{J}$, is a mathematical representation that captures how a vector field changes near a critical point. It plays a key role in classifying critical points by analysing their local behaviour. By studying the eigenvalues and eigenvectors of $\mathbf{J}$, we can understand how tangent curves behave around the critical point.

We classify attracting and repelling types of critical points (as shown in Fig S3) based on the eigenvalues of the Jacobian matrix. An attracting node is characterised by negative real parts ($R_1, R_2 < 0$) and zero imaginary parts ($I_1 = I_2 = 0$), whilst an attracting focus also has negative real parts ($R_1 = R_2 < 0$) but non-zero conjugate imaginary parts ($I_1 = -I_2 \neq 0$). A repelling node is defined by positive real parts ($R_1, R_2 > 0$) and zero imaginary parts ($I_1 = I_2 = 0$), whereas a repelling focus has positive real parts ($R_1 = R_2 > 0$) and non-zero conjugate imaginary parts ($I_1 = -I_2 \neq 0$) [13]. To identify these structures, we use the following parameters:

$$\text{det} = \lambda_1\lambda_2 = (R_1 + I_1 i)(R_2 + I_2 i)\,, \qquad \text{(S7 Eq)}$$

$$\text{trace} = \lambda_1 + \lambda_2 = (R_1 + I_1 i) + (R_2 + I_2 i)\,, \qquad \text{(S8 Eq)}$$

where 'det' represents the determinant of the Jacobian matrix and 'trace' represents the trace of the Jacobian matrix. For both attracting structures, det > 0 and trace < 0; for both repelling structures, det > 0 and trace > 0.

Fig S3: **Vector field structure of attracting and repelling critical points.** For each type, the characteristics of the Jacobian eigenvalues (real and imaginary parts), as well as the determinant and trace of the Jacobian matrix, are stated above the corresponding field structure.

S7 Moran's I

The Moran's I statistic quantifies the degree of spatial autocorrelation by analysing both the values and locations of observations. It assesses whether a variable exhibits spatial clustering, dispersion, or randomness, summarising spatial patterns within a dataset. The Global Moran's I index is calculated as follows [14]:

$$I = \frac{n}{\sum_i \sum_j w_{ij}} \times \frac{\sum_i \sum_j w_{ij} z_i z_j}{\sum_i z_i^2}\,, \qquad \text{(S9 Eq)}$$

where n is the number of observations; z_i is the standardised value of the observation at location i (commonly $z_i = y_i - \bar{y}$); y_i and y_j are values at locations i and j, respectively; $\bar{y}$ is the mean of all values; and w_{ij} represents the spatial weights matrix, defining the spatial relationship between pairs of locations.

Moran's I values range from -1 to $+1$:

- **Positive values** indicate spatial clustering, where similar values are near each other.
- **Negative values** suggest spatial dispersion, where high values are situated near low values, and vice versa.
- **Values close to zero** imply randomness, with no discernible spatial pattern in the data.

As a graphical tool, the Moran plot provides a valuable resource for understanding both global and local spatial autocorrelation. These plots take the form of scatter plots, where values of interest are plotted against spatial lag (as shown in Fig 7A) for each location [15].

The spatial lag variable represents weighted sums or averages of neighbouring values. The spatial lag characterises how a variable behaves near each location, essentially acting as a local smoother. This can be expressed in matrix notation as follows [14, 16, 17]:

$$y_{sl-i} = \sum_j w_{ij} y_j\,. \qquad \text{(S10 Eq)}$$

In Equation S10 Eq, y_{sl-i} represents the spatial lag for location i, calculated as the weighted sum of values at all other locations. As mentioned before, y_j is the value in location j, and w_{ij} is the entry in the i-th row and j-th column of a $\mathbf{W}$ spatial weights matrix. Since non-neighbours receive a weight of zero, y_{sl-i} effectively captures the weighted average of values at observation i's neighbours. Spatial lag of the vector magnitudes is calculated using spatial weights based on queen weights [18]. This weight reflects adjacency relationships using a binary variable that indicates whether a polygon shares an edge or a vertex with another polygon. The matrix expression for this weight is as follows:

$$W_{ij} = \begin{cases} 1, & \text{if regions } i \text{ and } j \text{ share an edge or vertex,} \\ 0, & \text{otherwise.} \end{cases} \qquad \text{(S11 Eq)}$$

S8 K-medoids clustering configuration

The clustering of municipalities and micro-regions, shown in Fig 5, was performed using the k-medoids algorithm (Partitioning Around Medoids, or PAM) [19]. The K-medoids algorithm was employed for clustering due to its robustness to noise and outliers and its compatibility with the precomputed Dynamic Time Warping (DTW) distance matrix, as it selects actual data points (medoids) as cluster centres. The optimal number of clusters was first estimated using the elbow method [20], which suggested $k = 4$ for the municipality and micro-region analyses (Fig S4). To robustly validate this estimate and evaluate cluster stability, the Gap Statistic was employed. The Gap Statistic [21] compares the total within-cluster dispersion of the observed data to that expected under an appropriate reference null distribution (i.e., data with no inherent clustering).

To compute dissimilarities, we employed DTW [22, 23], as commodity flow vectors are temporal sequences. DTW allows comparison of similarity patterns even when they are shifted or stretched in time, making it more appropriate than Euclidean distance, which assumes strict alignment. A distance matrix was constructed using DTW values, which then served as the input for the K-medoids algorithm.

Fig S4: **K-medoids clustering configuration.** **(A)** Cluster validity curve based on the within-cluster sum of squared distances (commonly referred to as the 'elbow method'), used to estimate the optimal number of clusters for municipalities and micro-regions. **(B)** Gap Statistic curve, used to validate the estimated number of clusters and assess clustering stability for municipalities and micro-regions, with a dashed line indicating the optimal number of clusters.

S9 Robustness of sinks and sources

In Fig S5, we demonstrate that most sinks and sources recalculated after area removal remain closely aligned with those identified using the complete dataset. As we progressively remove data from randomly selected areas, the impact is more pronounced on sources, as indicated by the steeper slope in Fig S5A. The less steeper slope in Fig S5B indicates that sinks are considerably less affected. Thus, our methodology demonstrates strong stability, with substantial effects only emerging after removing more than 50% of the data (> 75km).

S10 Limitations and generalisation of the method

A limitation of the proposed approach lies in the use of triangular interpolation, specifically its sensitivity to grid resolution. In areas with high-magnitude vectors, large grid cells can cause undersampling, as the linear approximation fails to capture fine-scale spatial variation. To reduce this error, grid cells must be small enough to resolve rapid changes in the data. In this study, the use of a uniform grid cell size likely introduced errors in regions of sharp variation. As a partial remedy, we imposed a maximum threshold on grid cell size based on the largest vector magnitude and the distance between OD nodes (Section S2). While this global heuristic reduced the most severe errors, it does not replace an adaptively refined grid that dynamically adjusts to local data properties, which would offer a more robust solution.

A limitation of this study arises when missing information is extensive within the system. As demonstrated in the robustness analysis (Robustness of vector fields section and Section S9), even when more than 50% of the area is excluded as missing data, the variation in direction estimates and critical point locations remains relatively small compared with the complete dataset. However, when data sparsity begins to affect model robustness, aggregating smaller regions into larger spatial units can provide an effective solution. Applying interpolation at a higher spatial scale helps to reduce both the amount and impact of missing data. The optimal balance between aggregation level and data completeness should be determined based on the study context; for example, street-level resolution may be appropriate for human mobility studies, whereas country-level aggregation may suit analyses of refugee

Fig S5: **Robustness of where the sinks and sources are calculated while removing a percentage of areas.** The y axis shows the relative distance between the original sinks and sources in comparison to the ones using the entire dataset.

migration. Focusing on a higher aggregation level can also help compensate for areas with limited or missing mobility information.

Our approach can be applied to any type of commodity mobility represented by an origin–destination matrix. Depending on the study objectives, various aggregation techniques, beyond simple averaging, can be employed to combine the outgoing mobility of each region and generate a final representative vector. The method also allows flexibility in interpolation strategies to handle missing information, such as adjusting spatial resolution or using alternative interpolation methods like radial basis function (RBF) interpolation [12], according to the data characteristics and research goals.

References

[1] geoBoundaries. geoBoundaries-BRA-ADM2-all; 2020. https://www.geoboundaries.org/countryDownloads.html.

[2] Brazilian Institute of Geography and Statistics (IBGE). RG2017_rgi_20180911; 2018. https://geoftp.ibge.gov.br/organizacao_do_territorio/divisao_regional/divisao_regional_do_brasil/divisao_regional_do_brasil_em_regioes_geograficas_2017/shp/RG2017_rgi_20180911.zip.

[3] Cressie N. Statistics for spatial data. John Wiley & Sons; 1993.

[4] Li Z, Zhu C, Gold C. Digital terrain modeling: principles and methodology. CRC press; 2004.

[5] Watson D, Philip G. Triangle based interpolation. Journal of the International Association for Mathematical Geology. 1984;16(8):779–795.

[6] Watson DF, Philip G. A refinement of inverse distance weighted interpolation. Geo-processing. 1985;2(4):315–327.

[7] Floater MS. Generalized barycentric coordinates and applications. Acta Numerica. 2015;24:161–214. doi:10.1017/S0962492914000129.

[8] geoBoundaries. geoBoundaries-IRQ-ADM0-all; 2021. https://www.geoboundaries.org/countryDownloads.html.

[9] Beggs JM, Timme N. Being critical of criticality in the brain. Frontiers in physiology. 2012;3:163.

[10] Tsallis C. Introduction to nonextensive statistical mechanics: approaching a complex world. vol. 1. Springer; 2009.

[11] Shannon CE. A mathematical theory of communication. The Bell system technical journal. 1948;27(3):379–423.

[12] Smolik M, Skala V. Vector field interpolation with radial basis functions. In: Proceedings of SIGRAD 2016, May 23rd and 24th, Visby, Sweden. 127. Linköping University Electronic Press; 2016. p. 15–21.

[13] Helman J, Hesselink L. Representation and display of vector field topology in fluid flow data sets. Computer. 1989;22(08):27–36.

[14] Rey S, Arribas-Bel D, Wolf LJ. Geographic data science with python. CRC Press; 2023.

[15] Kawano Y. workshop-python-spatial-stats; 2021. https://github.com/yohman/workshop-python-spatial-stats. Accessed 2025-05-10.

[16] Anselin L. Local indicators of spatial association—LISA. Geographical analysis. 1995;27(2):93–115.

[17] Darmofal D. Spatial analysis for the social sciences. Cambridge University Press; 2015.

[18] Berry BJL, Marble DF. Spatial analysis: a reader in statistical geography. Englewood Cliffs, NJ, Prentice-Hall. 1968;.

[19] Kaufman L, Rousseeuw PJ. Finding groups in data: an introduction to cluster analysis. John Wiley & Sons; 2009.

[20] Winarta A, Kurniawan WJ. Optimasi cluster k-means menggunakan metode elbow pada data pengguna narkoba dengan pemrograman python. JTIK (Jurnal Teknik Informatika Kaputama). 2021;5(1):113–119.

[21] Tibshirani R, Walther G, Hastie T. Estimating the number of clusters in a data set via the gap statistic. Journal of the royal statistical society: series b (statistical methodology). 2001;63(2):411–423.

[22] Ratanamahatana CA, Keogh E. Three myths about dynamic time warping data mining. In: Proceedings of the 2005 SIAM international conference on data mining. SIAM; 2005. p. 506–510.

[23] Sakoe H, Chiba S. Dynamic programming algorithm optimization for spoken word recognition. IEEE transactions on acoustics, speech, and signal processing. 2003;26(1):43–49.
