# Supplementary figures and images for: Vector fields as a framework for modelling the mobility of commodities
Source: PLoS One. 2026 Mar 13;21(3):e0340109. doi: 10.1371/journal.pone.0340109 (PMC12987471; doi:10.1371/journal.pone.0340109)

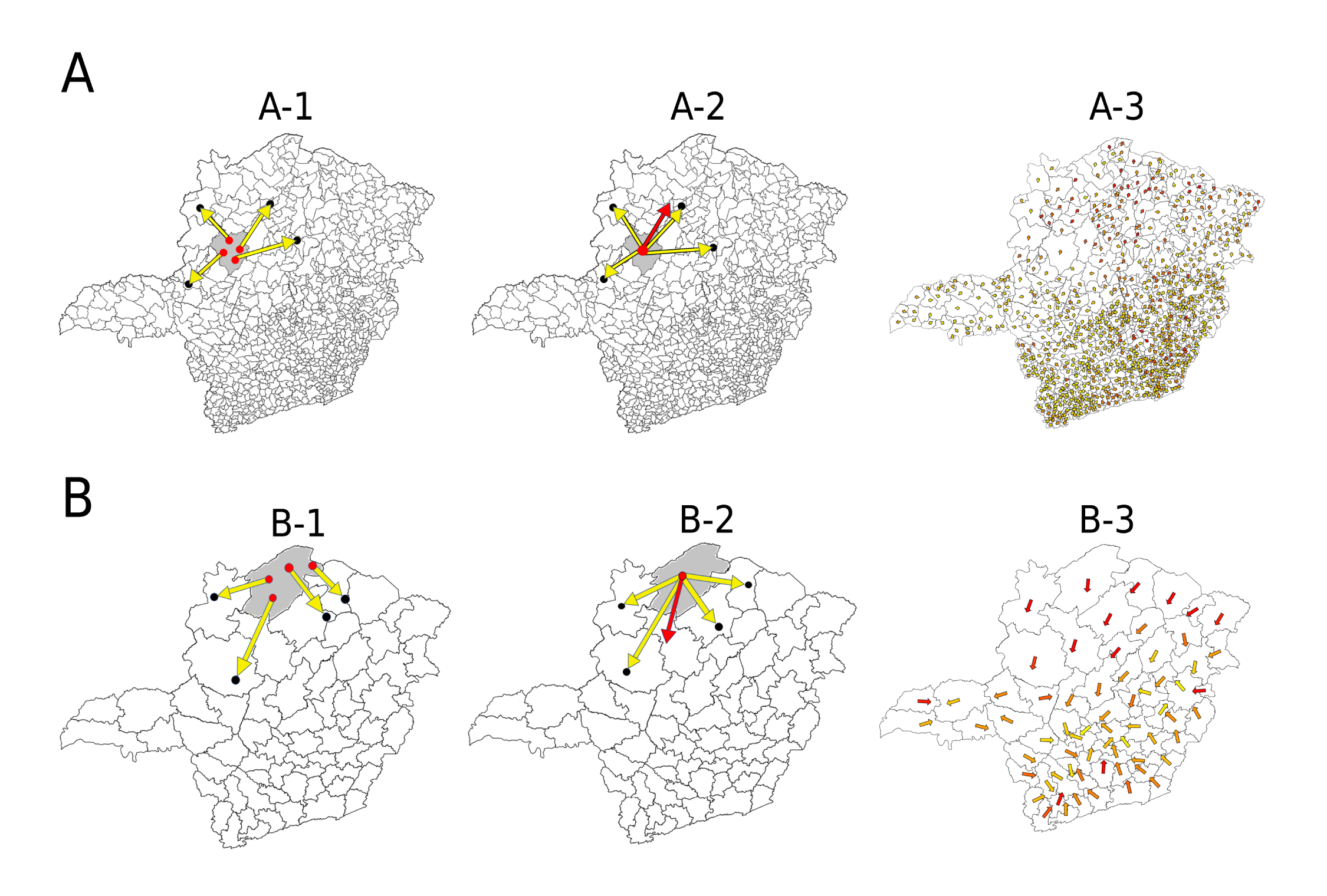

Supplement: S1 Fig — (A-1) and (B-1) illustrate trade flows originating from a grey part (municipality or micro-region) to others. (A-2) and (B-2) represent the transformation of these trades into vectors, drawn from the centre of the grey area to the centres of destination areas, and then aggregated into a single resultant vector (shown in red). (A-3) and (B-3) demonstrate complete vector fields, where interpolation has been used to estimate vectors for municipalities or micro-regions lacking trade data during the selected time window. Different colours indicate varying vector magnitudes. This approach is applied to a specific time window and geographic division but can be adapted to different temporal or spatial granularities depending on analytical needs. Base maps used in this figure are freely available (Panel A: [34]; Panel B: [35]). (TIFF) [file pone.0340109.s002.tiff]

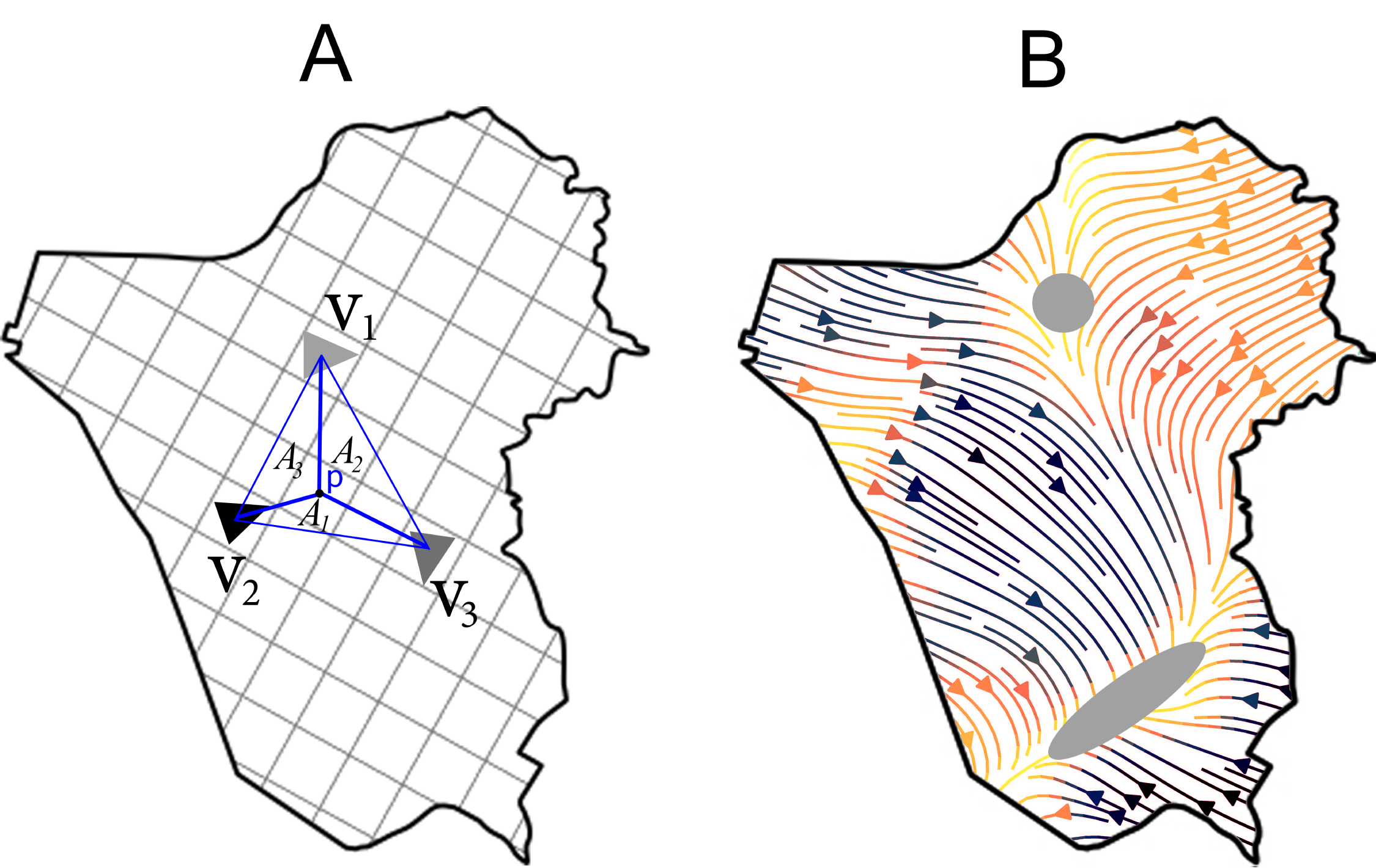

Supplement: S2 Fig — (A) Triangle-based interpolation method. To estimate vectors at specific locations within the triangulated mesh (generated using Delaunay triangulation on points with known vectors), we use a triangle-based interpolation technique. This method calculates the vector at a point by using the vectors at the three surrounding vertices. (B) Interpolated vector field. The resulting field displays interpolated vectors, with grey regions indicating critical points, which represent areas of attraction or repulsion. Colour variations reflect differences in vector magnitudes. The map used in this figure is freely available (not copyrighted) [31]. (TIFF) [file pone.0340109.s003.tiff]

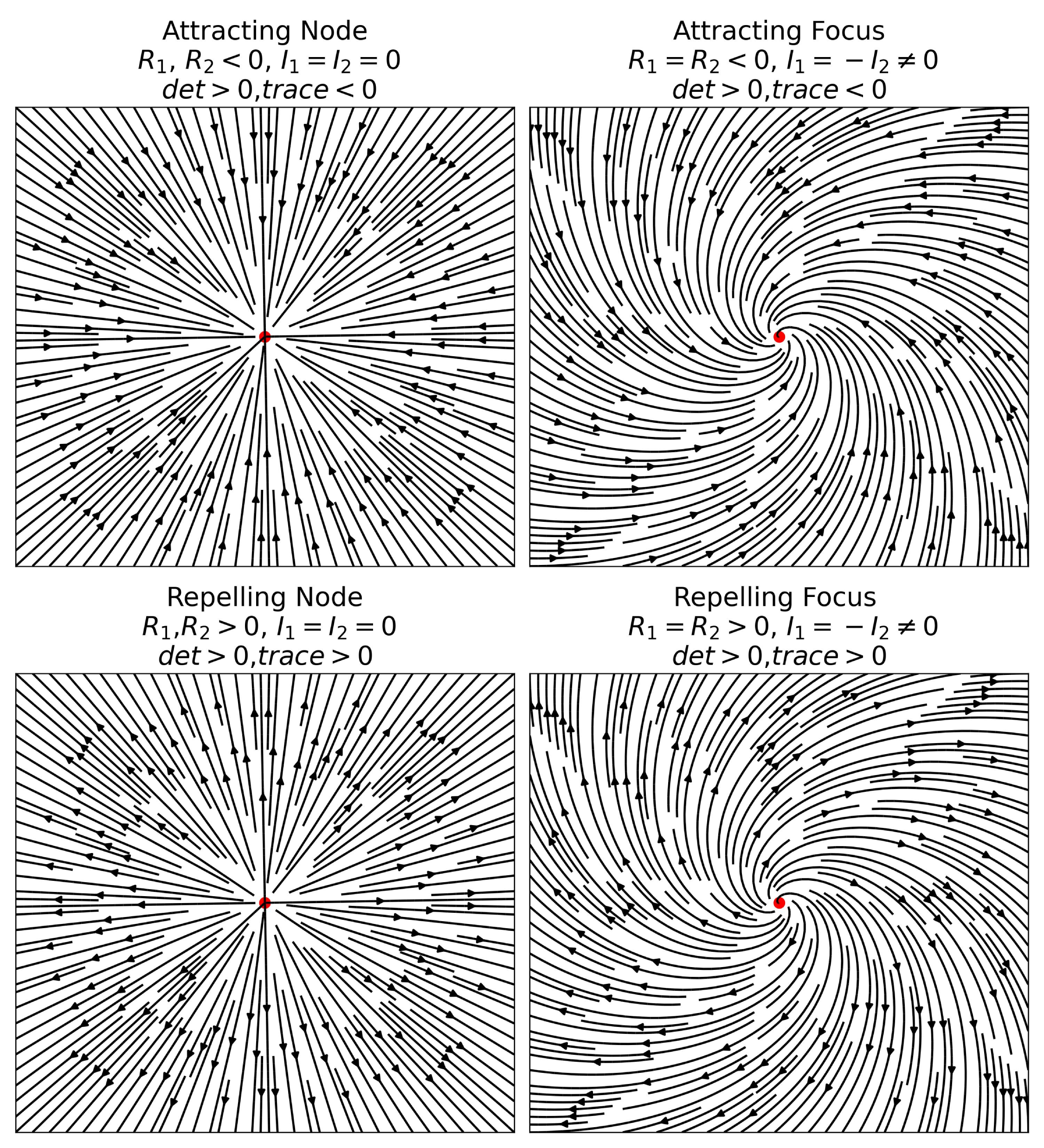

Supplement: S3 Fig — For each type, the characteristics of the Jacobian eigenvalues (real and imaginary parts), as well as the determinant and trace of the Jacobian matrix, are stated above the corresponding field structure. (TIFF) [file pone.0340109.s004.tiff]

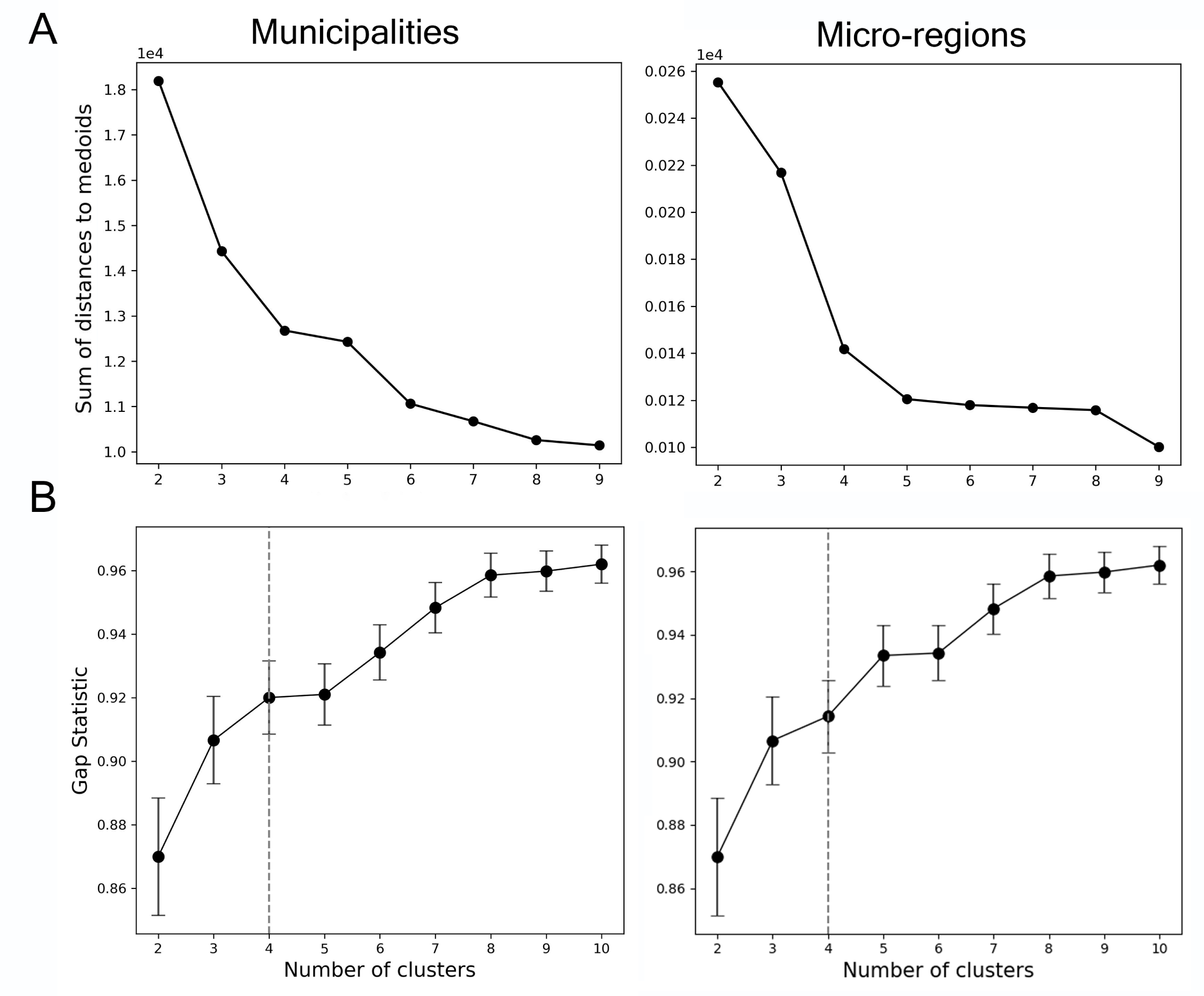

Supplement: S4 Fig — (A) Cluster validity curve based on the within-cluster sum of squared distances (commonly referred to as the ‘elbow method’), used to estimate the optimal number of clusters for municipalities and micro-regions.(B) Gap Statistic curve, used to validate the estimated number of clusters and assess clustering stability for municipalities and micro-regions, with a dashed line indicating the optimal number of clusters. (TIFF) [file pone.0340109.s005.tiff]

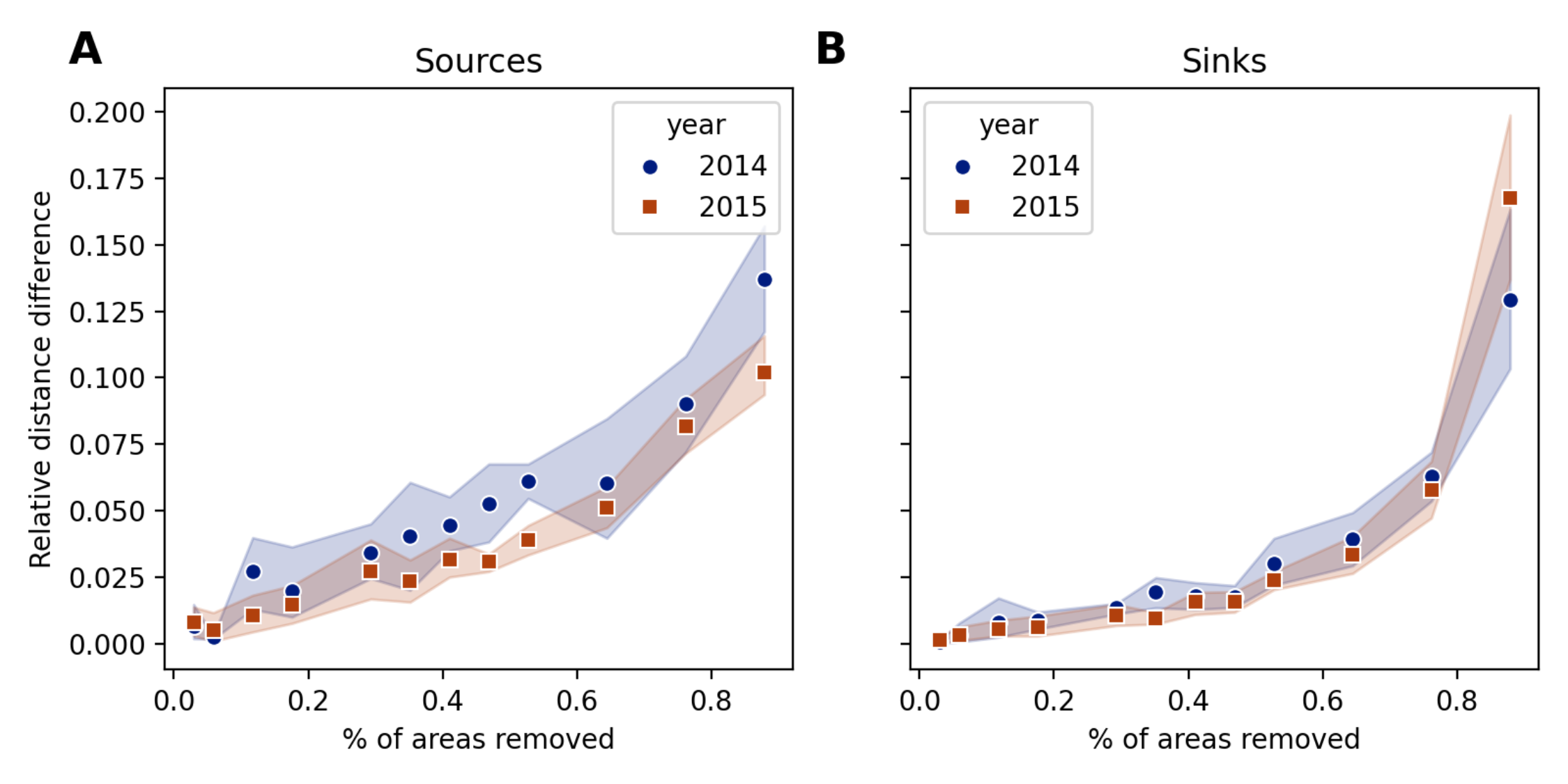

Supplement: S5 Fig — The y axis shows the relative distance between the original sinks and sources in comparison to the ones using the entire dataset. (TIFF) [file pone.0340109.s006.tiff]
